# Supplementary material for: A set of multi-entry identification keys to African frugivorous flies (Diptera, Tephritidae)
Source: Zookeys. 2014 Jul 24;(428):97–108. doi: 10.3897/zookeys.428.7366 (PMC4143993; doi:10.3897/zookeys.428.7366)
Supplement: Supplementary material 6 — Key to Ceratitis [file zookeys-428-097-s006.zip › SF6_ZooKeys_key to Ceratitis/key/SF6_ZooKeys_key to Ceratitis/Media/Html/Ceratitis capitata.htm]

Ceratitis (Ceratitis) capitata (Wiedemann)


***Ceratitis*** **(*Ceratitis*) *capitata*
(Wiedemann)**

*Tephritis capitata* Wiedemann, 1824: 55.

Body
length: 4.00 (3.45-4.60) mm; wing length: 4.12 (3.65-4.55) mm.

Male

Head.
Antenna yellow or more orange; first and second segment and base of third
segment sometimes darker. Third antennal segment twice as long as second
segment. Arista with short hairs, mainly on base and only distinct dorsally.
Frons convex to flat; yellow, sometimes with darker orange or orange-brown
patches including darker band near antennal implant, occasionally with faint
silvery shine; with short scattered hairs which are largely the same colour as
frons. Frontal and ocellar bristles black; lower orbital modified, stem pale
and shorter than arista with apical end dark and diamond-shaped; upper orbital
weakly developed, black. Face yellow-white. Genal bristle pale, genal setulae
pale or reddish, weakly developed. Postocellar and outer vertical pale.

Thorax.
Postpronotum white, with distinct black spot. Mesonotum: ground colour black,
microtrichiae pattern silvery with ashgrey shine, spots black except sutural
white spots, prescutellar white markings merged. Scapular setae pale. Scutellum
yellow-white, basally with two dark spots, separate or narrowly touching,
apically with three merged spots, only slightly incised. Anepisternum pale with
lower half darker yellow, pilosity variable but at least partly dark in lower
half. 

Legs
yellow; setation typical for subgenus, mainly pale especially on femora. Fore
femur posterodorsally with bush of longer orange-coloured hairs along entire
length, basally these hairs darker red or brown but not distinct black;
posteriorly hairs much shorter; ventral spines yellow-orange; anteroventral row
of hairs short and yellow-orange. Hind femur with longer hairs dorsally and
ventrally on apical fourth. 

Wing.
marginal band usually with clear and complete interruption, occasionally
narrowly or partly touching; cubital band free; medial band absent; crossvein
r-m at or near middle of discal cell. Vein R1 beyond or equal with crossvein
r-m. Orientation crossvein dm-cu variable.

Abdomen.
Yellow. Setation and banding typical for subgenus.

Female

As
in male except for the following characters: Third antennal segment in general
darker than in male. Frons sometimes with darker hairs; darker patches never as
outspoken as in male. Orbitals not modified, well developed. Genal setulae
darker and strongly developed. Anepisternum without darker pilosity. Legs
without feathering; ventral spines on fore femur sometimes partially dark. Oviscape
shorter than abdominal terga. 

(Description
after De Meyer, 2000)
